# In Vivo Detection of Tetrodotoxin in *Takifugu obscurus* Based on Solid-Phase Microextraction Coupled with Ultrahigh-Performance Liquid Chromatography–Tandem Mass Spectrometry

**DOI:** 10.3390/molecules27186122

**Published:** 2022-09-19

**Authors:** Hengli Meng, Shui Jiang, Yin Zhang, Yun Hu, Yuan Liu

**Affiliations:** 1 Department of Food Science & Technology, School of Agriculture & Biology, Shanghai Jiao Tong University, Shanghai 200240, China; 2 Shanghai Engineering Research Center of Food Safety, Shanghai 200240, China; 3 Key Laboratory of Meat Processing of Sichuan, Chengdu University, Chengdu 610106, China; 4 Yangzhou Center for Food and Drug Control, Yangzhou 225000, China

**Keywords:** tetrodotoxin, *Takifugu obscurus*, solid-phase microextraction, ultrahigh-performance liquid chromatography–tandem mass spectrometry

## Abstract

Pufferfish is nutritious and delicious, but the tetrodotoxin (TTX) that may exist in its body poses a serious safety hazard. It is important to use scientific and effective methods to detect the TTX in pufferfish, but most of the existing methods require complex pre-treatment steps and have sample lethality. The solid-phase microextraction (SPME) technology can be used for in vivo detection due to its advantages such as no solvent demand, simple operation, and fast detection speed. In this study, the GO-PAN@PNE SPME fibers were made via a dipping method, and their extraction effect was verified in the TTX aqueous and spiked fish. The established method has good reproducibility, and the limit of detection of TTX in pufferfish was 32 ng·g^−1^, and the limit of quantitation was 150 ng·g^−1^, which can meet the detection needs of pufferfish for safe consumption. This method was used to in vivo detect the *Takifugu obscurus* exposed to the TTX, to determine the content of TTX in the pufferfish muscle. The detection method established in this study can relatively quickly and easily realize the in vivo detection of TTX in the pufferfish, which can provide theoretical support for improvement in the food safety level of the pufferfish.

## 1. Introduction

Pufferfish is a general name for a group of poisonous economic fish species of Pelecypoda, containing more than 200 species, distributed in temperate, tropical, and subtropical regions. Pufferfish is rich in flavoring amino acids, nucleotides, and some flavor peptides, which form the unique flavor of pufferfish [1], so it is welcomed by consumers all over the world, especially in China, Japan and Korea. However, the possible presence of tetrodotoxin (TTX) in pufferfish poses a great threat to the safety of pufferfish consumption. Pufferfish poisoning incidents frequently occur all over the world, especially in Asian countries [2,3].

TTX is generally believed to be produced by marine bacteria and enriched in higher organisms such as pufferfish through the food chain and mostly accumulates in the liver and ovaries of pufferfish [4]. Tetrodotoxin is a neurotoxin that can selectively block sodium channels on the surface of muscle and nerve cell membranes when ingested, thereby blocking action potentials and causing nerve and muscle paralysis, which can lead to death in severe cases [5]. Furthermore, tetrodotoxin has high stability, which is difficult to be destroyed by normal cooking methods, and there is no targeted antidote or antitoxin after poisoning [6]. Therefore, it is very important to detect tetrodotoxin in pufferfish using scientific methods to avoid the occurrence of tetrodotoxin poisoning.

Traditionally, the detection of TTX relies on the mouse bioassay, immunoassay, and instrumental assay. The mouse bioassay is a traditional assay method that determines the TTX content based on the time of death of mice after the injection of sample extracts. Different mouse strains have different susceptibility to TTX [7]. The limit of detection of TTX using Kunming strain mice is 0.56 μg·g^−1^ [8]. The mouse bioassay is intuitive and has low costs, but it has a high detection limit, poor reproducibility, and no specificity. The principle of an immunoassay is the qualitative or quantitative analysis of the substance to be measured in the sample by the specific binding of antigen and antibody. The commonly used immunoassay for the detection of TTX is the enzyme-linked immunosorbent assay (ELISA) and the colloidal gold strip test [9]. The immunoassay method is highly sensitive, specific, and easy to operate. In instrumental assays, the level of TTX in the sample is obtained by extracting the TTX from the specimen with a specific solution and then measuring the TTX content with an instrument. The LC-MS method is a commonly used instrumental method for TTX detection, which has a low limit of detection and a good linear range [10,11]. All of the above methods can achieve the detection of TTX, and each has its advantages and disadvantages. However, most of the above methods require complex pre-treatment steps and have sample lethality, which destroys the higher commercial value of pufferfish. Therefore, it makes sense to find a rapid in vivo method for detecting TTX. The pre-treatment of samples often requires the biological sample to be killed and the corresponding solvent to be used for the extraction of the substances to be tested, which is not only a complex and cumbersome procedure but also unethical.

The solid-phase microextraction (SPME) technique, first proposed by Professor Janusz Pawliszyn in Canada in 1990 [12], is a solvent-free extraction method. It is a non-exhaustive extraction method, based on the partitioning equilibrium of the substance to be measured in the sample and the extraction coating, a phase equilibrium process. This technique was selected as one of the six greatest ideas in the field of analytical science in the 1990s, together with electrospray ionization and capillary electrophoresis [13]. Since the sampling process of SPME does not require the use of solvents and is less damaging to the analytical system, it can be applied to in vivo testing (in vivo SPME). In vivo SPME does not require extraction, centrifugation, purification, and other processes; rather, it simplifies these steps into one step, which greatly shortens its detection time and significantly improves the sampling efficiency; moreover, in vivo SPME technology is less damaging to the analyte system and is more suitable for the analysis of high-value or rare plants and animals such as pufferfish. In addition, in vivo SPME can more accurately reflect the contents and changes in analytes in living organisms, which is conducive to the tracking study of individual organisms [14]. Given these advantages of in vivo SPME technology, in vivo SPME has been used in studies about plants [15], animals [16,17], insects [18], and even humans [19]. 

In this work, we applied the SPME technique to the detection of TTX, constructed a rapid analysis method for TTX with a self-made SPME fiber, and attempted to use the fiber for the in vivo detection of TTX, to realize the hazard identification and risk assessment of TTX and provide theoretical support for improving the food safety of pufferfish.

## 2. Results and Discussion

### 2.1. Preparation and Characterization of SPME Fibers

**Figure 1:**
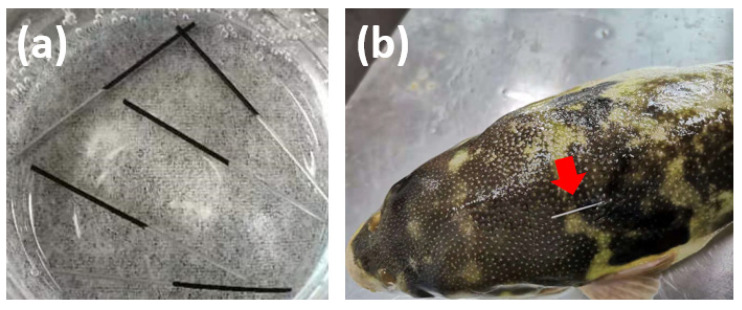
The GO-PAN@PNE fibers made via dipping method (**a**) and the in vivo sampling in the dorsal–epaxial muscle of living pufferfish using the SPME fiber (**b**).

GO-PAN@PNE fibers with brown color and uniform coating thickness were successfully prepared via a dipping method (Figure 1). In recent years, many new materials are used as novel SPME fiber coatings, including some carbon materials such as graphene oxide (GO) [20]. GO is an oxide of graphene and has been used for the SPME of various polar substances [21,22,23]. After oxidation treatment, GO still maintains the layer structure of graphite, and oxygen-containing groups that are highly hydrophilic and have a large specific surface area, such as carboxyl, hydroxyl, and epoxy groups, are introduced on each layer of graphene monoliths [24]. The aforementioned advantages make the GO a potential material with good adsorption capacity to TTX. The PAN is used as a biocompatible polymer in biomedical applications, such as dialysis and ultrafiltration. Its high levels of chemical and mechanical stability allow PAN to be used as an ideal stationary phase fixative [15]. The NE, formed when N-methyl is removed from epinephrine, is a catecholamine neurotransmitter that shares a similar oxidative polymerization process with the common neurotransmitter dopamine [25]. Polynoradrenaline (PNE) is the product of a polymerization reaction, and it is capable of sheath wrapping on its own and possesses good biointerfacial properties. In this study, PNE was used to improve the extraction performance and corrosion resistance of the SPME fibers. 

During heat treatment, the carboxyl group on GO sheets nucleophilically attacks the carbon of the cyano group to initiate the anionic cyclization of the PAN molecular chain, which in turn connects GO and PAN to form a more stable membrane structure [26]. Graphene oxide–polyacrylonitrile composites have applications in the fields of electrochemistry, dye removal, and VOC adsorption [27,28,29,30] but have been rarely used in direct immersion SPME.

**Figure 2:**
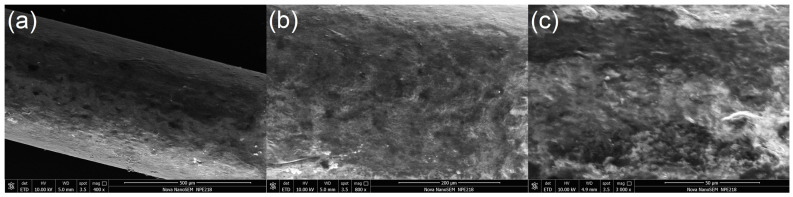
SEM images of the GO-PAN@PNE fiber in different magnifications: (**a**) 400×; (**b**) 800×; (**c**) 3000×).

A Nova Nano-SEM 450 field-emission scanning electron microscope (FESEM, FEI, USA) was used to characterize the fibers. The fiber surfaces were observed at magnifications of 400, 800, and 3000 (at higher magnifications, the coating was damaged by the high-energy electron beam). As shown in Figure 2, at lower magnifications, the overall surface of the fiber coating showed a smooth and uniform morphology without large bumps, depressions, and agglomerates, and this uniform distribution facilitated the reproducibility of the fibers. At higher magnification, it can be observed that the surface of the fiber gradually became rougher and appeared folded and porous. The DMF was evaporated from the coating solution, and the remaining PAN and GO were fixed on the surface of the fiber, which formed a stable structure. Such a structure was beneficial to increasing the contact area and improving the loading capacity of the fiber, which enhanced the extraction performance.

### 2.2. In Vitro Evaluation of SPME Fibers

**Figure 3:**
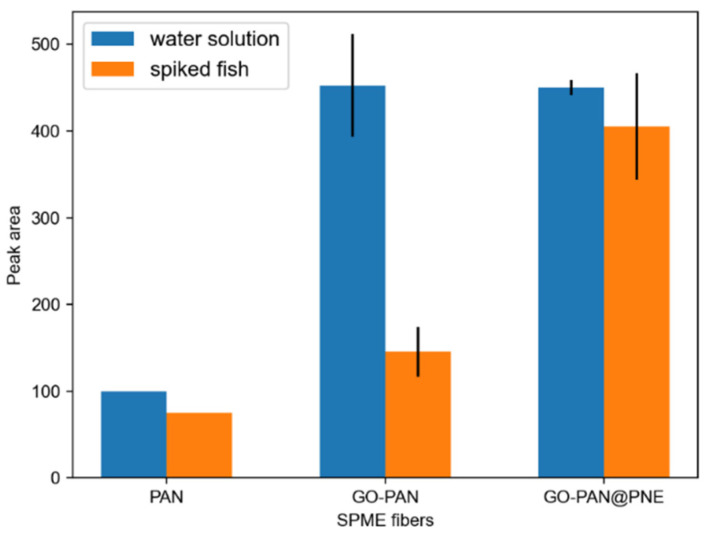
In vitro evaluation of extraction performance of PAN, GO-PAN, and GO-PAN@PNE fibers.

The extraction of a 1 μg·mL^−1^ aqueous solution of TTX and 1 μg·g^−1^ spiked fish was performed using blank PAN fibers, GO-PAN fibers, and GO-PAN@PNE fibers, respectively, to evaluate the extraction performance of the self-made fibers. As shown in Figure 3, the extraction performance of the SPME fibers with the addition of GO was significantly improved, compared with the blank PAN fibers, because GO has a large specific surface area and strong adsorption to small molecules. Furthermore, the surface of GO contains oxygen-containing groups that are highly hydrophilic and can produce hydrogen bonding and induce forces on tetrodotoxin molecules during the extraction process. It can also be observed that the extraction amount of TTX in the fish meat was lower than that in the aqueous solution when the fibers were not wrapped with the PNE bionic sheath, while the GO-PAN@PNE fibers with the addition of the PNE bionic sheath had better extraction performance in the fish meat. This is because the fish meat samples not only contained TTX but also biomolecules such as proteins, which may reduce the loading capacity of the fibers by binding to the surface and competing with TTX for the adsorption sites during the extraction process. At the same time, the biomolecules in the elution solvent reduced the ionization efficiency of the sample during the LC-MS/MS analysis. These biomolecules that adhere to the fiber surface are not easily cleaned off, thus reducing the loading capacity of the fibers for the next use. PNE with good resistance to biocorrosion can inhibit the attachment of biomacromolecules such as proteins to the surface and enhance the extraction performance of the fibers in fish. Meanwhile, the interface of the PNE sheath layer is hydrophilic and does not hinder the binding of TTX to the coating.

### 2.3. Method Optimization

The results of SPME were related to various factors. The extraction time, elution solvent, and elution time were used as variables in turn to investigate the effects of these factors on the extraction amount and to select the best experimental method for subsequent evaluation and in vivo SPME extraction.

#### 2.3.1. Extraction Time

**Figure 4:**
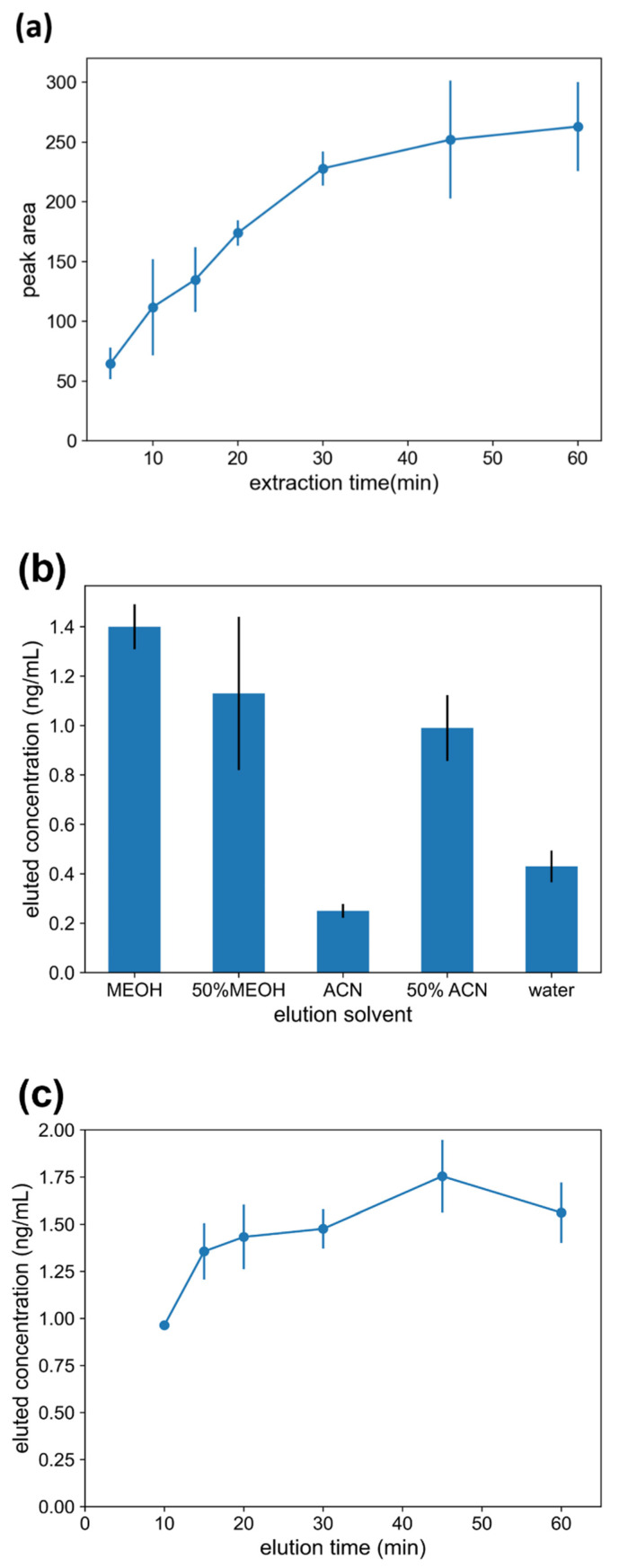
Optimization of SPME method using GO-PAN@PNE fibers in spiked fish samples: (**a**) extraction time; (**b**) elution solvent; (**c**) elution time.

The SPME process is essentially an equilibrium of the analytes between the matrix and the extraction phase, which requires a certain amount of time to reach equilibrium. Extraction was carried out in the fish meat samples with 1 μg·g^−1^ TTX in the time range of 5–60 min. As shown in Figure 4a, the extraction efficiency increased with the extraction time and had little change after 30 min; thus, the extraction time was set as 30 min for subsequent experiments. This extraction time is close to some previous in vivo SPME studies [15,17,31].

#### 2.3.2. Elution Solvent

The elution solvent greatly affects the efficiency of analyte removal from the fibers. Pure water, 50% (*v*/*v*) methanol/water, pure methanol, 50% (*v*/*v*) acetonitrile/water, and pure acetonitrile were selected as elution solvents for the experiments to select the most suitable solvent for elution. It could be observed that pure methanol had the best effect on the elution of TTX, as shown in Figure 4b, so methanol was selected as the best elution solvent, which is consistent with the previous research [31].

#### 2.3.3. Elution Time

Similar to the extraction process, the elution process also needs a certain time to reach equilibrium. As shown in Figure 4c, the best extraction performance was obtained when the elution time was 30 min.

### 2.4. Method Evaluation

The proposed in vivo SPME extraction method was further evaluated with the linear range, the limit of detection (LOD), the limit of quantitation (LOQ), and reproducibility. Eight concentration gradients of 5, 10, 50, 100, 250, 500, 750, and 1000 ng·mL^−1^ (or ng·g^−1^) were determined in the TTX aqueous and TTX spiked fish meat, respectively. As shown in Table 1, the linear range was good in the concentration range of 150–1000 ng·g^−1^, and the *R*^2^ values were 0.9955 in the aqueous and 0.9895 in the spiked fish. The LOD and LOQ of the method were calculated based on the signal-to-noise ratios of 3 and 10, respectively. The LOD and LOQ of the fibers in the TTX aqueous solution were lower than those in the TTX spiked fish meat, which is probably because the biomolecules in the fish samples bound to the fiber surface and competed with TTX for the adsorption sites during extraction, leading to a decrease in the specificity of the fibers for TTX. 

The LOD for the fish analyzed with this method is far lower than that of the traditional mouse biological method (0.56 μg·g^−1^) [8]. However, the LOD of this method is higher than that of the instrumental analysis using the liquid-phase extraction method [10,11]. Liquid-phase extraction could completely extract the TTX in the sample by grinding the sample to minced status and using the solvent. The SPME technology is an in vivo extraction method that belongs to non-exhaustive extraction, and only a small part of the TTX in the sample was extracted by using the SPME fibers. The lethal dose of TTX for humans is about 10,000 MU (about 2 mg) [3]. A concentration of 2 μg·g^−1^ of TTX in a 1 kg pufferfish is required to reach lethal levels, so the LOQ of 150 ng·g^−1^ of this method can fully meet the needs of food safety testing for pufferfish.

The reproducibility of the proposed method was determined in a 1000 ng·mL^−1^ TTX aqueous solution and 1000 ng·g^−1^ spiked fish. As shown in Table 1, the inter-fiber RSD values of the SPME fibers in the aqueous solution and spiked fish meat were 4.63 and 8.82, respectively, indicating that the uniformity of the SPME fibers’ preparation was good. However, the intra-fiber reproducibility was not very ideal, especially in the spiked fish. Although PNE was used as the biological sheath in the fiber preparation process to improve the anti-biological contamination ability of the fibers, the extraction stability of a single fiber in spiked fish meat still decreased with the increase in extraction time, possibly because the PNE sheath was scraped off in the fish flesh. The biocorrosion resistance and service life of the fibers need to be improved.

### 2.5. In Vivo SPME Extraction

The results of in vivo SPME are shown in Table 2. On the day of the start of the exposure experiment, one of the pufferfish (No. 1) was detected using SPME extraction, and no TTX was detected, which indicated that the pufferfish was suitable for the exposure experiment. After the pufferfish were exposed to TTX for 7 days, the dorsal muscles of the pufferfish surviving in good condition were extracted in vivo using the self-made SPME fibers to obtain the level of TTX. The results of fish No. 4 and No. 6 were higher than the LOD (32 ng·g^−1^) but lower than the LOQ (150 ng·g^−1^), which can indicate the presence of TTX in the fish muscle but cannot be precisely quantified. While the TTX concentration of fish No. 5 was below the LOD, it could not be determined that fish No. 5 contained TTX. During the feeding process of the pufferfish, the phenomenon of fish death occurred due to long transportation time, the replacement of the feeding environment, and changes in the feeding water quality. There were several fish that died 2 days after exposure (No. 2), and 6 days after exposure (No. 3). In order to explore the content of TTX in the body of pufferfish after death, we also performed in vivo SPME extraction on the dead pufferfish, using the method described in Section 3.6 with the anesthesia step omitted. 

The result of the exposure experiment demonstrated that pufferfish could accumulate TTX in the muscles when eating TTX-contained food, and the TTX contents of the different pufferfish individuals were different. Furthermore, TTX could not be detected in the muscle of those pufferfish that were not fed with TTX-contained food. These results of the exposure experiment could be explained by the exogenous theory in the TTX source theory [4]. Generally speaking, when the TTX concentration of pufferfish is lower than 100 MU·g^−1^ (about 20 μg·g^−1^), it can be considered that pufferfish are non-toxic or low-toxic to the human body [17], so all the pufferfish in this research were considered non-toxic to humans. 

At the same time, we also found that after the fish died, the level of TTX in the muscles significantly increased, and this increase might even be close to 10 times. As shown in Table 2, the TTX contents in the dorsal muscles of two dead pufferfish (No. 2 and No. 3) were higher than that of the living fish. Even the fish that had been fed TTX for only 1 day (No. 2) had a higher TTX content in the muscle after death than the living pufferfish (No. 4, 5, and 6) that had been fed for 7 days. The reason for this phenomenon may be that, after the fish died, the TTX in the blood, liver, gonads, and other parts with high content of TTX diffused into the muscle, resulting in a substantial increase in the content of TTX in the muscle. Therefore, when cooking pufferfish, it should be handled in time after the death of the fish to avoid the increase in TTX in the muscles.

## 3. Materials and Methods

### 3.1. Chemicals and Materials

The species of the pufferfish used in this experiment is *Takifugu obscurus*, which was purchased from Jiangsu Zhongyang Company (Nantong, China). The weight of the pufferfish was about 250g. Medical stainless steel wires (0.5 × 2.5 cm) were purchased from Guangdong Longxin Co. (Zhongshan, China). Graphene oxide (GO) powder (>98 wt%) was purchased from 3A Chemicals Co. (Shanghai, China). Polyacrylonitrile (PAN, average Mw 85000), norepinephrine (NE, 98%), and glacial acetic acid (analytical purity) were purchased from Macklin Co. (Shanghai, China). N,N-dimethylformamide (DMF, analytical purity), tetrodotoxin (≥99%), and methanol (chromatographic grade) were purchased from Aladdin Reagent Co. (Shanghai, China). Tris(hydroxymethyl)amine (TRIS, ≥99%) was purchased from Solarbio Co. (Beijing, China). And ultrapure water was prepared by the water purifier purchased from Chengdu Haokang Technology Co. (Chengdu, China).

### 3.2. Preparation of SPME Fibers

The GO-PAN@PNE fibers used in this study were prepared via a simple dipping method, with the stainless steel wires as the substrates. 

#### 3.2.1. Preparation of Coating Solution

Firstly, 0.1 g of PAN solid was added to 0.8 g of DMF in a 1.5 mL centrifuge tube, and the suspension was heated at 90 °C for 1 h to fully dissolve the PAN solid and form PAN glue. Then, 20 mg of GO powder was added to 250 μL of DMF, and the mixture was ultrasonicated for 1 h to make the GO uniformly distributed; then, it was added to the PAN glue and stirred well to form a coating solution. 

#### 3.2.2. Dipping Process of Fibers

The medical stainless steel wires were cut into small segments with a length of 2.5 cm and ultrasonicated in concentrated hydrochloric acid for 10 min to activate the steel wire surface. Additionally, the GO-PAN@PNE fiber was prepared via a dipping method: One end of the treated stainless steel wire was dipped vertically into the coating solution and taken out slowly; then, the fiber was heated at 90 °C for 3 min to volatilize DMF and fix the coating. The dipping and heating steps were repeated several times so that the coating was wrapped as evenly as possible on the steel wire, and the final thickness of the coating was about 50 μm. 

#### 3.2.3. Addition of PNE Bionic Sheath

A certain amount of NE powder was dissolved in a mixed solvent of Tris buffer (10 mM, pH = 8.5) and methanol (1:1, *v*/*v*) to prepare the NE solution (2.0 mg·mL^−1^). Afterward, the prepared GO-PAN fibers were thoroughly submerged in the NE solution for 24 h. 

### 3.3. Characterization of SPME Fibers

The self-made GO-PAN@PNE SPME fibers were cut to the appropriate size (about 1 cm) and attached to the sample stage with a conductive adhesive. A Nova Nano-SEM 450 field-emission scanning electron microscope (FESEM, FEI, Hillsboro, OR, USA) was used to observe the surface morphology of the fibers.

### 3.4. Extraction Performance Evaluation

In addition to the final GO-PAN@PNE fibers, blank PAN fibers without GO addition and the GO-PAN fiber without the PNE sheath layer were prepared as references. The TTX aqueous (1 μg·mL^−1^) and TTX spiked fish (1 μg·g^−1^) were used as samples to conduct the in vitro SPME extraction. The extracted TTX amount was used to evaluate the performances of these three fibers and to confirm the role of GO and PNE in the extraction. The sample preparation and extraction method for the spiked fish were as follows: A few *Takifugu obscurus* purchased from Jiangsu Zhongyang Company (Nantong, China) were dissected, and 5 g of their dorsal muscles were ground into minced meat and then added into 10 mL glass sample bottles. Then, 5 mL of the aqueous solution of TTX was added to prepare a spiked fish sample of TTX, which was used for the in vitro SPME extraction. The pretreated fibers were inserted vertically into the glass bottles containing the spiked fish sample to ensure that the coating was completely immersed in the samples. The extraction process was controlled for a certain period. After extraction, the fiber was rinsed with ultrapure water for 3 s and then dried with a Kimwipe paper towel. The extracted TTX was eluted from the fibers with 250 μL of elution solvent for a certain time. The elution solution was analyzed via the LC-MS/MS analysis. The in vitro extraction was repeated to optimize the extraction time, elution time, and elution solvent.

### 3.5. Exposure Experiment

The pufferfish were raised in a fish tank containing 40 L of oxygenated dechlorinated tap water and fed with eel meal containing TTX. The exposure dose of TTX was 2 MU·g^−1^ body mass·day-1 (each pufferfish was fed with about 100 μg of TTX every day; MU: mouse unit, 1 MU represents the average amount of toxin that kills a male mouse weighing 20 g within 30 min after intraperitoneal administration, and 1 MU is equivalent to about 0.2 mg). The pufferfish exposure experiment lasted for 7 days. The feeding condition was controlled during the exposure period. The water temperature was controlled at about 25 °C with an air conditioner. The water was continuously oxygenated with an air pump to ensure the oxygen content of the water body. A filter device was used to filter the residual bait and feces. Nitrifying bacteria were added to establish a nitrification system. Additionally, 0.5% of NaCl was added to maintain water osmotic pressure. During the exposure experiment, the in vivo SPME extraction was performed to determine the TTX content in the pufferfish.

### 3.6. In Vivo SPME Extraction

The procedure of in vivo SPME extraction in the fish muscle was similar to previous research [17,31]. The pufferfish was picked up from the water and submerged in a 0.1% eugenol (*v*/*v*) aqueous solution until its body was unbalanced. A medical syringe needle was inserted into the side of the dorsal muscle near the fin of the pufferfish and then was withdrawn to obtain an approximately 1.5 cm deep pinhole. Afterward, the SPME fiber was inserted into the pinhole to extract the TTX in the muscle, as shown in Figure 1b. During the extraction process, the pufferfish was placed back into the clean water to make it free to swim. After the extraction, the pufferfish was picked up from the water again, and the SPME fiber was withdrawn from the muscle. Finally, the TTX was eluted by the solvent from the SPME fiber for the instrumental analysis to obtain the TTX content in the pufferfish muscle.

### 3.7. Instrumentation (UPLC-MS/MS)

The instrumental analysis was based on a UPLC system coupled to a triple-quadrupole tandem mass spectrometer (Nexera LC30AD&SCIEX SelexION Triple Quad 5500 System). An ACQUITY BEH HILIC column (2.1 × 100 mm, 1.7 μm) was used for separation, and the column temperature was 40 °C, and the injection volume is 2 μL. Gradient elution was applied by using water with 0.1% formic acid as mobile phase A and ACN with 0.1% formic acid as mobile phase B. The initial gradient of 5% A was kept unchanged for 0.5 min, then ramped to 50% A in 2.5 min and ramped to 95% A in 1 min, and kept for 2 min, then decreased to 5% A in 1 min, and kept for 1.9 min. The flow rate was set at 0.4 mL·min^−1^, and the total analysis time for each sample was 8 min. 

The mass spectrum parameters were as follows: electrospray ionization (ESI); the positive ionization mode; curtain gas 35 psi; ion spray voltage 5500 V; desolvation temperature 600 °C; nebulizing gas 55 psi; auxiliary heating gas 55 psi; analysis of the samples via multiple reaction monitoring (MRM). The transition was monitored with the positive ion mode at *m*/*z* 320.1/162.0, and the detailed mass spectrometry parameters are shown in Table 3. 

**Figure 5:**
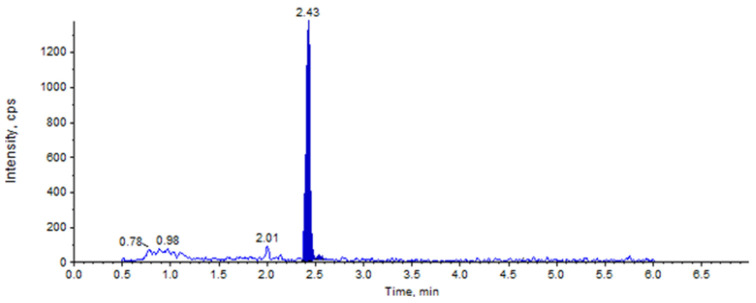
UPLC-MS/MS chromatogram of TTX standard.

In the UPLC-MS/MS results, the characteristic ion and retention time (around 2.43 min) were used for qualitative analysis, and the characteristic peak areas for quantitative analysis. The UPLC-MS/MS chromatograms of the TTX standard are shown in Figure 5. 

## 4. Conclusions

In this study, a detection method of TTX in pufferfish was established based on in vivo SPME extraction and UPLC-MS/MS. The GO-PAN fibers were fabricated through a dipping method, followed by biomimetic PNE sheath modification. The self-made SPME fibers could successfully extract TTX in both the spiked fish samples and living pufferfish, and the LOD of the proposed method was 32 ng·g^−1^, which can meet the requirement for safe consumption of pufferfish. The exposure experiment demonstrated that the pufferfish could accumulate TTX in the muscles when eating TTX-contained food, and the TTX level in the muscles of the dead pufferfish significantly increased up to 10 times that of the living pufferfish. The established method could quickly and easily detect the TTX content in pufferfish in vivo without damage to the living pufferfish. This study provides theoretical support for improvement in the food safety level of pufferfish.

## Figures and Tables

**Table 1:** Linear range, limit of detection (LOD), limit of quantitation (LOQ), and reproducibility.

Samples	LOD	LOQ	Linear Range	*R* ^2^	Reproducibility (RSD, %, *n* = 6)	
					Intra-Fiber	Inter-Fiber
TTX aqueous	11.8 ng·mL^−1^	81.3 ng·mL^−1^	100–1000 ng·mL^−1^	0.9955	13.0	4.63
TTX spiked fish	32 ng·g^−1^	150 ng·g^−1^	150–1000 ng·g^−1^	0.9895	36.1	8.82

**Table 2:** In vivo SPME results of TTX.

Pufferfish Number	Exposure Days	Calculated TTX Content (ng·g^−1^)
1	0	0
2	2	141
3	6	480
4	7	91
5	7	25
6	7	53

Pufferfish No. 1, 2, and 3 died before testing, and pufferfish No. 1 had not been exposed to TTX.

**Table 3:** Mass spectral parameters of TTX.

Precursor Ion (*m*/*z*)	Product Ion (*m*/*z*)	Declustering Potential (V)	Collision Energy (eV)
320	302	80	40
	162	80	35

## Data Availability

Not applicable.

## Sample Availability

Samples of the compounds are not available from the authors.
